# Alterations of White Matter Integrity Related to the Season of Birth in Schizophrenia: A DTI Study

**DOI:** 10.1371/journal.pone.0075508

**Published:** 2013-09-27

**Authors:** Stéphanie Giezendanner, Sebastian Walther, Nadja Razavi, Claudia Van Swam, Melanie Sarah Fisler, Leila Maria Soravia, Jennifer Andreotti, Simon Schwab, Kay Jann, Roland Wiest, Helge Horn, Thomas Jörg Müller, Thomas Dierks, Andrea Federspiel

**Affiliations:** 1 Department of Psychiatric Neurophysiology, University Hospital of Psychiatry, University of Bern, Bern, Switzerland; 2 University Institute of Diagnostic and Interventional Neuroradiology, Inselspital and University of Bern, Bern, Switzerland; King’s College London, United Kingdom

## Abstract

In schizophrenia there is a consistent epidemiological finding of a birth excess in winter and spring. Season of birth is thought to act as a proxy indicator for harmful environmental factors during foetal maturation. There is evidence that prenatal exposure to harmful environmental factors may trigger pathologic processes in the neurodevelopment, which subsequently increase the risk of schizophrenia. Since brain white matter alterations have repeatedly been found in schizophrenia, the objective of this study was to investigate whether white matter integrity was related to the season of birth in patients with schizophrenia. Thirty-four patients with schizophrenia and 33 healthy controls underwent diffusion tensor imaging. Differences in the fractional anisotropy maps of schizophrenia patients and healthy controls born in different seasons were analysed with tract-based spatial statistics. A significant main effect of season of birth and an interaction of group and season of birth showed that patients born in summer had significantly lower fractional anisotropy in widespread white matter regions than those born in the remainder of the year. Additionally, later age of schizophrenia onset was found in patients born in winter months. The current findings indicate a relationship of season of birth and white matter alterations in schizophrenia and consequently support the neurodevelopmental hypothesis of early pathological mechanisms in schizophrenia.

## Introduction

Schizophrenia is a psychiatric disorder with a plethora of mysteries that prevents the assessment of an exact aetiology [1]. Although genetic factors are involved substantially in the risk of developing schizophrenia, environmental factors have recently received much attention [2]. Notably, there is a consistent phenomenon of a 5–8% excess of winter births in patients with schizophrenia, which is replicated throughout the world. The exact mechanisms causing a season of birth effect have not yet been identified, since season of birth merely acts as a proxy indicator for prenatal exposure to seasonally varying harmful environmental factors [3]. Epidemiological, clinical and imaging findings provide evidence for the neurodevelopmental hypothesis of schizophrenia, which suggests that the exposure to harmful factors during foetal maturation may detrimentally affect neurodevelopmental processes resulting in continuous brain and behavioural changes throughout childhood and adolescence, which eventually lead to a higher risk of schizophrenia [4].

Schizophrenia is accompanied by significant, progressive anatomical brain changes, appearing in early adulthood [5]. A finding of interest has been the demonstration of microstructural white matter (WM) alterations in patients with schizophrenia using diffusion tensor imaging (DTI) [6]–[8]. Reduced white matter integrity has been observed as early as first-episode schizophrenia in widespread brain regions [9]. It has been proposed that the downregulation of oligodendrocyte and myelin-related genes may underlie WM alterations in schizophrenia [10]. Typically, brain WM starts to develop during the late first trimester of foetal maturation and is completed by the age of 2 years [11]–[13], followed by a myelination process lasting until early adulthood [14], [15].

Previous studies have already probed for a relationship between season of birth and brain anatomical properties in schizophrenia indicating a ventricular enlargement in winter and spring births, but no other anatomical associations [16]–[21]. Notably, these studies used either computerized tomography or magnetic resonance imaging. However, DTI has not yet been implemented to test the effect of season of birth on WM properties in schizophrenia. Since diffusion is highly anisotropic in brain white matter, reduced anisotropy of water diffusion has been proposed to reflect compromised white matter integrity [22]. Interestingly, after prenatal exposure to viral infections in rodents, WM integrity abnormalities and myelin-related gene alterations were detected [23]. Therefore, according to the neurodevelopmental hypothesis of schizophrenia, it is conceivable that white matter properties in humans may be affected by harmful environmental factors during foetal maturation, which are related to the season of birth. Consequently, in the present study an association between WM integrity and season of birth is hypothesized in patients with schizophrenia. According to previous anatomical findings, we expected to find WM integrity reductions in patients born in winter relative to those born in summer [16]. However, WM integrity may also be compromised during other birth seasons, since the timing of the seasonal factor is unknown and its effects may differ depending on developmental processes affected. For healthy control subjects such an association is not expected, since the lack of psychopathology suggests normal neurodevelopment. To test the current hypotheses, WM integrity of schizophrenia patients and healthy controls was measured with DTI and analysed with tract-based spatial statistics (TBSS).

The purpose of this study was to clarify a possible association between season of birth and WM integrity. Specifically, we hypothesized a relationship between WM integrity and season of birth in patients with schizophrenia and that this association would not be observed in healthy controls.

## Materials and Methods

### Ethics Statement

Procedures were approved by the local ethics committee (of the Kanton Bern, Switzerland: KEK Bern, 192-05, 064-05, 196-09) and are in accordance with the Declaration of Helsinki. First we explained in detail to all patients and healthy controls the planed study. This was done orally and on the basis of the written description of the study and included the aims and procedures of the study. All subjects were explicitly informed that participation was voluntary and could be declined at any time without any reason and without being subjected to any disadvantage. The patients’ capacity to provide informed consent was assessed and confirmed by their treating psychiatrist who was independent from the study. All participants provided written informed consent prior to the beginning of the examination. The participants received a copy of their signed consent and were not paid for their participation.

### Participants

Thirty-four patients with schizophrenia who were admitted to the University Hospital of Psychiatry in Bern and 33 healthy controls who did not differ in age and gender participated in this study. The healthy controls were recruited among the hospital employees of various professions. Inclusion criteria for the patients were Diagnostic and Statistical Manual of Mental Disorders, Fourth edition diagnoses of schizophrenia, schizoaffective disorder or schizophreniform disorder [24]. Out of the 34 patients, 28 were diagnosed with schizophrenia, 4 were diagnosed with schizophreniform disorder, and 2 were diagnosed with schizoaffective disorder. The exclusion criteria for both groups were any serious medical or neurological condition, drug abuse other than nicotine, history of electroconvulsive treatment or head injury with a subsequent loss of consciousness. Additional exclusion criteria of controls were history of any psychiatric disorder and first degree relatives suffering from schizophrenia spectrum disorders. Symptom severity was assessed by the Positive and Negative Symptom Scale (PANSS) [25]. The estimated current equivalent dosage of antipsychotics was calculated according to Woods [26]. Other environmental factors which may play a role in the development of schizophrenia such as migration, cannabis use and minority status were extracted from the medical history of each patient. Minority status was based on the patient’s ethnicity. In order to probe for the seasonal effects on brain white matter, the current study adopted the seasonal differentiation found in literature for the group assignment, which showed significant birth excesses during November and May [3]. Consequently, subjects born from November to May were assigned to the winter half year group (winter-born subjects), whereas the summer half year group (summer-born subjects) comprised subjects born from June to October. In addition, a systematic comparison of different half-year classifications that comprised all 6 possible combinations of equally classifying the year into 2 halves were conducted in order to verify the specificity of the season-of-birth effect within the DTI analysis.

**Figure 1:**
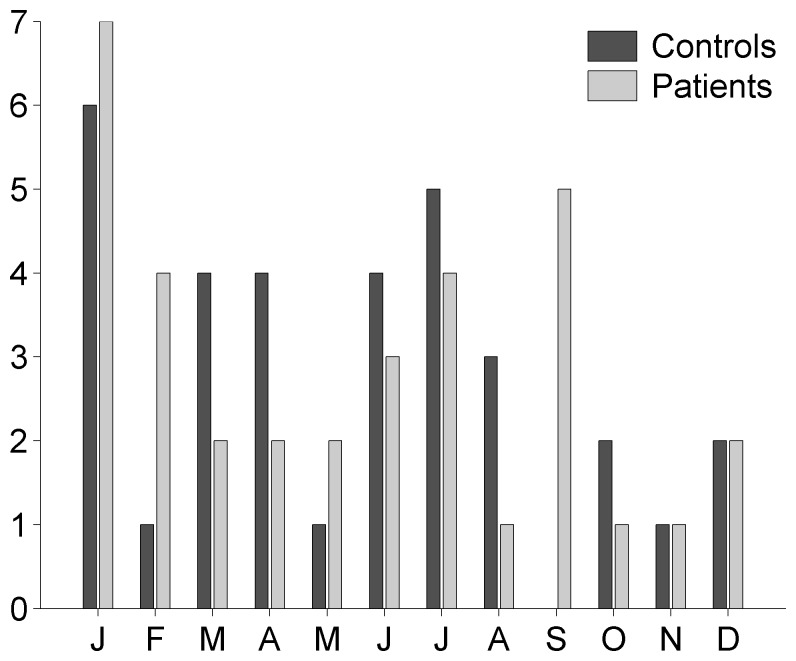
Distribution of the months of birth across the year for patients and controls. The x-axes of the bar plot displays the months from January to December, each abbreviated by the first letter. The y-axis reports the frequency of births.

The demographic and clinical characteristics of the participants are shown in Table 1. Patients and healthy controls were not different in age, gender distribution or season of birth. No significant differences were observed for age, gender, migration, cannabis use or minority status ratio among winter- and summer-born patients and winter- and summer-born healthy controls. Because clinical and demographic variables were not normally distributed, non-parametric tests were used to assess differences between groups with significance set at *p*<0.05 using R software package [27]. Furthermore, a MATLAB Toolbox for Circular Statistics was employed to analyse the birth distribution of patients and healthy controls across the year and their correlates with demographic and clinical variables with significance set at *p*<0.05 [28]. Circular statistics confirmed the lack of group differences in birth distribution during the year (Figure 1). Additionally, correlation analyses of birth dates with demographic and clinical variables demonstrated an absence of significant relationships, except for the age of schizophrenia onset, which indicated an increase in the age of schizophrenia onset for patients born in January and February (see Supporting Information, Figure S1).

**Table 1:** Demographic data of patients with schizophrenia and controls, separated for seasons of birth.

Category	Controls			Patients		
	Total	Winter-born	Summer-born	Total	Winter-born	Summer-born
*N*	33	19	14	34	20	14
Age (years)	33.2 (±8.9)	31.15 (±8.17)	36.07 (±9.41)	33.65 (±9.89)	34.00 (±9.45)	33.14 (±10.83)
Age range	21–53	21–52	22–53	20–66	20–55	22–66
Gender (M/F)	(16/17)	(11/8)	(5/9)	(18/16)	(12/8)	(6/8)
Age at onset (years)				25.63 (±7.15)	26.51 (±8.85)	24.28 (±4.24)
Duration of illness (years)				8.22 (±8.79)	7.75 (±8.11)	8.86 (±9.91)
PANSS-P				14.29 (±6.14)	14.70 (±7.15)	13.71 (±4.50)
PANSS-N				18.15 (±7.91)	17.90 (±8.58)	18.50 (±7.12)
PANSS-T				60.91 (±18.96)	60.10 (±19.18)	62.07 (±19.30)
CPZ (mg)				506.41 (±87.84)	417.75 (±279.12)	633.07 (±678.89)
Migration (yes/no)				(8/26)	(6/14)	(2/12)
Minority status (yes/no)				(12/22)	(8/12)	(4/10)
Cannabis use (yes/no)				(17/17)	(10/10)	(7/7)

Except for age range, gender, migration, social adversity and cannabis use, values are represented as mean ± standard deviation. M, male; F, female; PANSS, Positive and Negative Syndrome Scale; PANSS-P, subscale for positive symptoms; PANSS-N, subscale for negative symptoms; PANSS-T, total score of PANSS, CPZ, chlorpromazine equivalents.

### Imaging Methods

DTI images were acquired for all subjects using a 3-Tesla Magnetom Trio TIM system (Siemens Erlangen, Germany) using a 12-channel array head coil. Spin-echo planar imaging sequence was performed with two 180° pulses and a repetition time/echo time of 6,500/96 ms with a matrix of 128×128, a field of view of 256×256 mm^2^, 52 slices, a slice thickness of 2 mm, a gap thickness of 0 mm, a pixel bandwidth of 1396 Hz/pixel and N = 2 averages, 4 b-values of 0 s/mm^2^ and a b-value of 1,300 s/mm^2^ along 42 non-collinear directions. All images were measured in the axial plane parallel to the anterior/posterior commissure line.

### DTI Processing

For the pre-processing of DTI images we implemented the Centre for Functional Magnetic Resonance Imaging of the Brain’s diffusion toolbox, part of the Centre for Functional Magnetic Resonance Imaging of the Brain software library (FSL, http://www.fmrib.ox.ac.uk/fsl) [29]. The original data was corrected for the effects of head movement and eddy currents. Subsequently, a brain extraction tool was used to remove all non-brain parts of the image [30]. From these images, fractional anisotropy (FA) values were calculated by fitting a tensor model to the data at each voxel. Tract-Based Spatial Statistics (TBSS, part of FSL) pipeline was used for further pre-processing of FA images [29] and included the following steps; All subjects’ FA data were aligned to a 1 mm×1 mm×1 mm Montreal Neurological Institute (MNI) standard space using as a target the FMRIB58 FA standard-space image. The alignment was performed by the non-linear registration tool FMRIB’s Non-Linear Image Registration Tool [31], [32], which uses a B-spline representation of the registration warp field [33]. Next, a mean FA image was created and thinned in order to create a mean FA skeleton that represented the centres of all tracts common to the group. A lower FA threshold of 0.2 was used to prevent the inclusion of non-skeletal voxels [34]. Each subject’s aligned FA data was then projected onto this skeleton, and the resulting data was subjected to voxel-wise cross-subject statistics.

### Statistical Data Analysis

Statistical analysis was carried out with TBSS, which is based on a non-parametric approach using permutation test theory with a standard general linear model (GLM) design matrix [34]. Age and gender as well as PANSS total scores, CPZ and age at onset were entered as covariates of no interest into the analyses. The GLM examined the main effect of season and group, as well as a season by group interaction. Pair-wise differences between groups, and also between subgroups (e.g. summer-born controls versus summer-born patients) were calculated in accordance to a study with a similar design [35]. The permutation testing was performed by the FSL Randomise program using 5,000 Monte Carlo random permutations to generate random permutation maps. This approach allows for inference on the statistical maps when the null distribution is unknown and offers a solution to the problem of multiple testing [36]. Using this setup voxel-wise differences between groups were then assessed, testing the significance corrected for multiple comparisons at *p*<0.05. Results which were uncorrected for multiple comparisons are reported in the Supporting Information (see Table S1 and Figure S2). A threshold-free cluster enhancement option was employed in order to enhance the cluster-like structures in images without having to define an initial cluster-forming threshold. From the results of the voxel-wise analyses, the skeletal regions showing significant effects were located and labelled anatomically by mapping the FWE-corrected statistical map to the Johns Hopkins University (JHU)-ICBM-DTI-81 WM labels atlas and the JHU-WM tractography atlas in MNI space [37], [38]. For the comparison of FA differences between groups, mean FA values were extracted from the significant clusters within the different WM regions for each subject. Only clusters greater than 100 voxels per WM region were displayed.

## Results

### Significant Main Effect of Season of Birth

**Figure 2:**
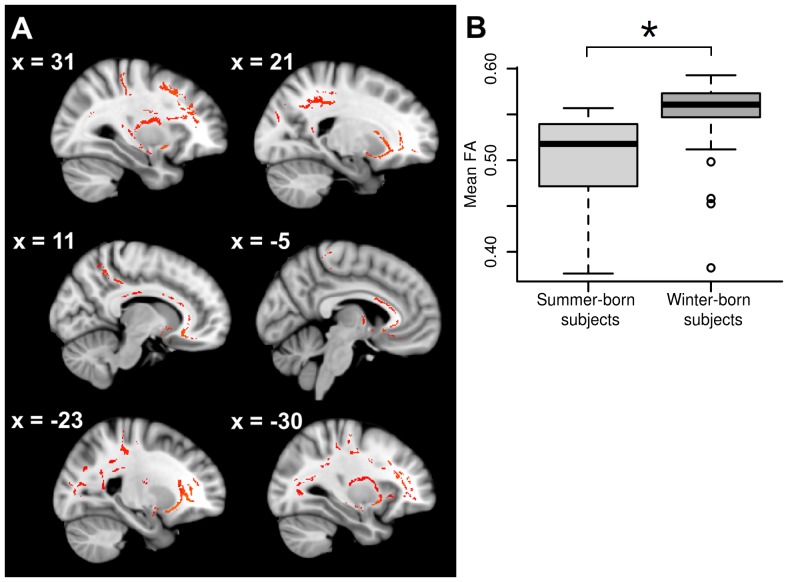
Significant main effect of season of birth. (A) Summer-born subjects have lower FA values than winter-born subjects in the regions indicated in red. (B) The plot shows the mean FA values in summer and winter-born subjects within the significant TBSS clusters of the main effect of season highlighted in red. The TBSS images show results at *p*<0.05 corrected for multiple comparisons.

A significant main effect of season was reported by the GLM applied in TBSS in widespread white matter regions (see Figure 2). Significantly decreased FA values were found in summer-born subjects (including healthy controls and patients) compared to winter-born subjects (*p<*0.05). The anatomical regions comprised sections of the corpus callosum, the internal capsule, the corona radiata, the posterior thalamic radiation, the sagittal striatum, the external capsule and the superior longitudinal fasciculus. For an overview of the size and mean FA values of the significant clusters, see Table 2.

**Table 2:** Main effect of season of birth for FA values.

Location	Centre of gravity (MNI coordinates)			Cluster size	Mean FA (±SD) of all subjects	
	x	y	z		Summer-born	Winter-born
Genu of corpus callosum	3	−4	25	920	0.74 (±0.07)	0.78 (±0.06)
Body of corpus callosum	3	−4	25	760	0.74 (±0.08)	0.78 (±0.06)
Splenium of corpus callosum	−3	−43	20	585	0.78 (±0.06)	0.82 (±0.04)
Anterior limb of internal capsule	19	13	6	275	0.58 (±0.06)	0.62 (±0.05)
Anterior limb of internal capsule	−16	10	5	492	0.53 (±0.04)	0.58 (±0.05)
Retrolenticular part of internal capsule	32	−27	5	203	0.60 (±0.07)	0.65 (±0.06)
Anterior corona radiate	21	30	0	699	0.53 (±0.05)	0.57 (±0.05)
Anterior corona radiate	−20	31	2	889	0.51 (±0.06)	0.56 (±0.06)
Superior corona radiate	27	−10	28	318	0.53 (±0.05)	0.57 (±0.05)
Superior corona radiate	−24	−12	29	133	0.60 (±0.06)	0.64 (±0.06)
Posterior corona radiate	22	−37	34	193	0.50 (±0.06)	0.54 (±0.05)
Posterior corona radiate	−25	−41	28	275	0.51 (±0.05)	0.56 (±0.05)
Posterior thalamic radiation	−32	−59	7	547	0.61 (±0.07)	0.67 (±0.05)
Sagittal stratum	41	−31	−9	364	0.58 (±0.08)	0.63 (±0.06)
Sagittal stratum	−40	−29	−9	233	0.59 (±0.04)	0.64 (±0.06)
External capsule	30	1	−2	714	0.46 (±0.05)	0.51 (±0.04)
External capsule	−29	4	0	738	0.52 (±0.05)	0.57 (±0.05)
Superior longitudinal fasciculus	38	−21	29	557	0.54 (±0.05)	0.58 (±0.05)
Superior longitudinal fasciculus	−36	−18	29	662	0.55 (±0.05)	0.59 (±0.05)

MNI, Montreal Neurological Institute; FA, fractional anisotropy; SD, standard deviation.

### Main Effect of Group

The GLM indicated a significant main effect of group exclusively when no correction for multiple comparisons was applied. Patients with schizophrenia showed a reduction in FA values compared to healthy controls (*p uncorrected*<0.05) in the bilateral superior longitudinal fasciculus, bilateral inferior-frontal fasciculus, in the bilateral forceps major and in the bilateral cingulum (see Figure S2 and Table S1).

### Interaction of Group and Season of Birth

**Figure 3:**
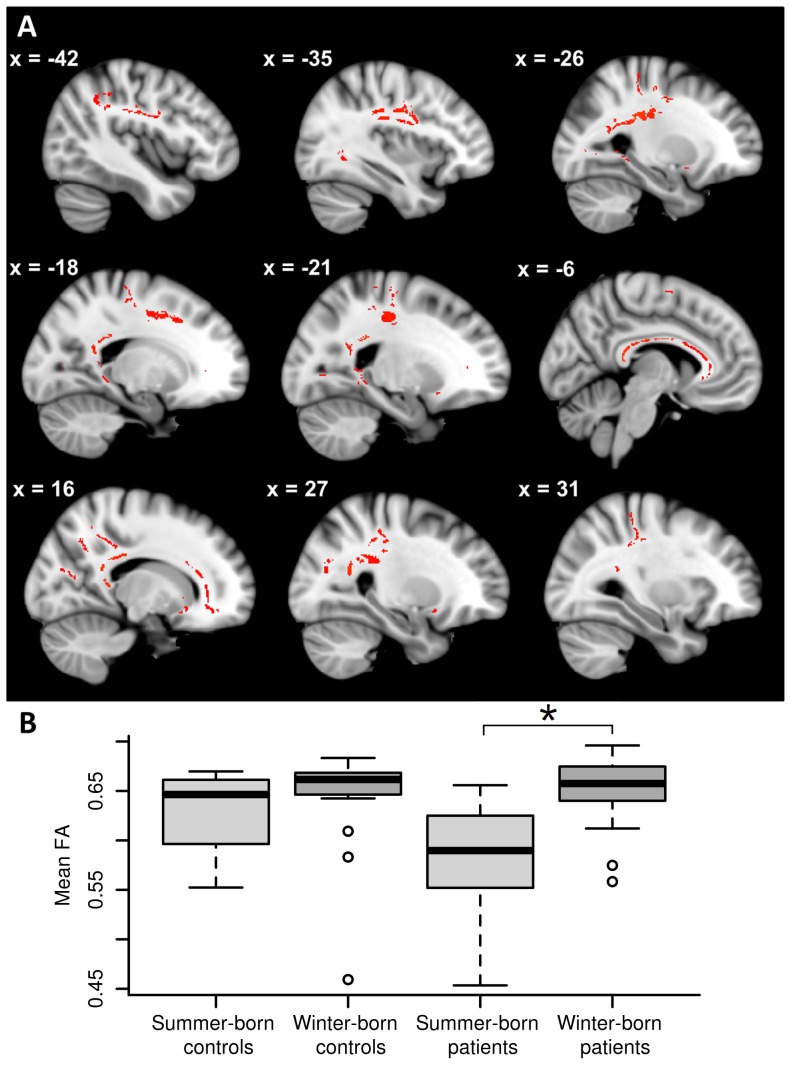
Interaction of group and season of birth. (A) Summer-born patients with schizophrenia showed reduced FA values compared to winter-born patients with schizophrenia within the areas indicated in red. (B) The plot shows the mean FA values within the significant TBSS clusters highlighted in red in summer and winter-born patients. For the purpose of comparison, mean FA values extracted from the same areas indicated in red were additionally displayed for summer and winter-born controls. The TBSS images show results at *p*<0.03 corrected for multiple comparisons.

The GLM revealed that summer-born patients with schizophrenia had significantly lower FA values than winter-born patients (*p<*0.05). As can be seen in Figure 3, the regions of decreased FA in summer-born patients relative to winter-born patients are predominantly located within the genu, body, and splenium of the corpus callosum. Furthermore, there were significant differences in parts of the inferior fronto-occipital fasciculi on both sides, the uncinate fasciculi on both sides, the right anterior corona radiata, the left posterior cingulum near the hippocampus, the posterior corona radiata in both hemispheres, the left posterior thalamic radiation, parts of the corticospinal tracts on both sides, the superior longitudinal fasciculi on both sides, and the forceps major. For an overview of the size and mean FA values of the significant results see Table 3. Systematically comparing different half-year classifications demonstrated significant results within patients but not within healthy controls. In particular, lower FA values were found for patients born between June and November compared to those born between December and May (*p*<0.05), as well as significantly lower FA for patients born between May and October compared to those born between November and April (*p*<0.05). No other significant interactions were found between group and season of birth when correcting for multiple comparisons. Notably, healthy controls displayed no areas of significantly altered FA values between different seasons of birth. For significant results which are uncorrected for multiple comparisons see the Supporting Information (see Table S1 and Figure S2). Except for age being negatively correlated with FA values, the voxel-wise analysis detected no significant associations between the duration of illness, the age at schizophrenia onset, CPZ or the PANSS total scores and FA values.

**Table 3:** Interaction of group and season of birth shows reduced FA values in summer-born patients compared to winter-born patients with schizophrenia.

	Centre of gravity (MNI coordinates)			Cluster Size	Mean FA (±SD) of patients	
Location	x	y	z		Summer-born	Winter-born
Genu corpus callosum	2	26	8	808	0.73 (±0.07)	0.81 (±0.04)
Body of corpus callosum	0	−4	25	1149	0.74 (±0.08)	0.81 (±0.04)
Splenium of corpus callosum	0	−40	19	1318	0.79 (±0.06)	0.85 (±0.03)
Anterior corona radiata	17	33	−1	227	0.55 (±0.06)	0.62 (±0.04)
Superior corona radiata	−23	−18	36	155	0.53 (±0.06)	0.59 (±0.05)
Posterior corona radiata	25	−39	28	422	0.50 (±0.05)	0.56 (±0.05)
Posterior corona radiata	−26	−39	25	293	0.53 (±0.06)	0.58 (±0.05)
Posterior thalamic radiation	−30	59	7	195	0.60 (±0.06)	0.66 (±0.04)
Inferior fronto-occipital fasciculus	−29	−59	5	197	0.54 (±0.09)	0.61 (±0.08)
Inferior fronto-occipital fasciculus	25	−39	19	396	0.52 (±0.06)	0.58 (±0.06)
Corticospinal tract	−22	−23	45	614	0.51 (±0.05)	0.57 (±0.04)
Corticospinal tract	23	−30	39	209	0.49 (±0.05)	0.55 (±0.05)
Cingulum near hippocampus	−20	−45	0	191	0.49 (±0.06)	0.56 (±0.03)
Superior longitudinal fasciculus	−36	−19	29	584	0.47 (±0.05)	0.54 (±0.04)
Forceps major	1	−50	15	859	0.71 (±0.05)	0.77 (±0.04)
Uncinate fasciculus	19	21	−10	185	0.44 (±0.04)	0.50 (±0.04)

MNI, Montreal Neurological Institute; FA, fractional anisotropy; SD, standard deviation.

## Discussion

We report a significant main effect of season of birth on white matter integrity which has not been previously noted. This significant main effect of season demonstrated that summer-born subjects had significantly lower FA values relative to winter-born subjects. The interaction of group by season of birth indicated that WM integrity impairments were higher within summer-born patients than within summer-born healthy controls relative to their winter-born counterparts. Furthermore, our findings indicated impaired WM integrity in schizophrenia patients relative to healthy controls. Although these findings are uncorrected for multiple comparisons they are broadly consistent with previous DTI studies [39]–[42]. In addition, a correlation of birth date and illness onset was demonstrated, indicating a later age of disease onset for patients born in winter months (see Supporting Information, Figure S1).

In previous studies, a birth excess in winter-born patients has repeatedly been found [3]. Harmful environmental factors are thought to affect neurodevelopment in perinatal stages and thus increase the risk of schizophrenia for winter-born subjects [43]. Furthermore, some studies have observed a ventricular enlargement in winter-born patients with schizophrenia relative to patients born in the remainder of the year suggesting detrimental effects by perinatal seasonal factors [19]. According to these observations and the neurodevelopmental hypothesis of schizophrenia, we expected WM integrity impairments in winter-born schizophrenia patients relative to the patients born in the reminder of the year. However, the current DTI study displayed structural white matter impairments in patients born in summer relative to patients born in winter. Consequently, this finding suggests that the seasonal birth excess in winter and WM integrity impairments in schizophrenia are unrelated.

The exact mechanisms that account for the observation of reduced WM integrity in patients born in summer are beyond the scope of this study. Since the season of birth is regarded as a proxy indicator for prenatal exposure to harmful factors, the current findings are still in line with the neurodevelopmental hypothesis of schizophrenia [4]. According to this hypothesis, the prenatal exposure to harmful factors may affect subsequent neurodevelopment resulting in an increased risk of schizophrenia [44]. It is suggested that abnormal brain development may start as early as late first or early second trimester and trigger further pathologic mechanisms [4]. This is evidenced by neuroanatomical irregularities already present at disease onset such as ventricular enlargement, grey and white matter changes in patients with schizophrenia [45], [46]. Consequently, the current findings of a relationship between season of birth and WM properties support the hypothesis of perinatal events involving a seasonal factor and subsequent pathologic brain development in schizophrenia [47].

As mentioned in the introduction, previous neuroimaging findings have already revealed a relationship between ventricular enlargement and season of birth, but not for other morphological measures [16]–[21]. Particularly, patients with schizophrenia born in winter-spring months had an increased risk of ventricular enlargement compared to patients born in the rest of the year. An animal study using immune activation in different gestational stages corroborated this finding showing a marked enlargement of lateral ventricles at adult stage, without affecting total white and grey matter volumes [48]. These findings contributed to the notion of an anatomical specificity of seasonally harmful environmental factors affecting neurodevelopment [19]. However, the current findings may add to the previous relationship between season of birth and ventricular enlargement because it is suggested that using DTI, more subtle changes of WM properties can be detected [22]. As mentioned in the introduction, animal models have already shown that the exposure to viral infections during foetal maturation resulted in subsequent white matter alterations, such as glial fibrillary acidic protein immunoreactivity [49] and abnormal expression of myelination gens which were detected using DTI [23]. As in rodents, human WM development starts during early foetal maturation and may thus be comparably affected by prenatal exposure to harmful factors [12].

The interaction of group by season of birth indicated that FA values in summer-born subjects relative to winter-born subjects were more reduced within patients than in healthy controls. As already mentioned, the current findings are unexpected regarding the previous findings of a ventricular enlargement in winter-born schizophrenia patients [20]. Nevertheless, this reported dissociation of season of birth and related anatomical alterations may indicate the importance of the timing of exposure to harmful factors during foetal maturation. Animal studies have demonstrated that middle or late prenatal exposure to infections were dissociable regarding brain and behavioural pathology [50]–[52]. Since the exact timing of action of the seasonal factor is unknown, the current results of reduced WM integrity in summer-born patients may merely indicate that WM properties and ventricular enlargement may be the result of exposure to harmful factors during different stages of perinatal development. Furthermore, the current observation of a later age at schizophrenia onset in winter-born patients together with previous findings of enduring negative symptoms for schizophrenia patients born in summer months contribute to the notion of differential effects of a seasonal factor in the aetiology of schizophrenia [53]. Interestingly, negative symptoms have been found to correlate with WM integrity in schizophrenia patients [54]. Consequently, the current results might indicate a differential effect of the timing of exposure to prenatal harmful factors during foetal maturation.

The main effect of season of birth showed that WM integrity was reduced in summer-born subjects relative to winter-born subjects. This effect was driven by the patients group showing significantly decreased WM integrity in summer-born patients relative to winter-born patients. However, this effect was also observed in healthy controls when correction for multiple comparisons was omitted. Since WM integrity changes are unspecific for schizophrenia and have been found in different neurologic and psychiatric diseases as well as in healthy aging [55]–[57], it is conceivable that it may vary in a small degree in healthy controls as well. Interestingly, the season of birth has already been related to cognitive and physical characteristics in healthy controls. Notably, a study which analysed the cognitive and physical outcome of healthy children depending on their season of birth revealed a superior cognitive and physical development in winter and spring-born children compared to summer and autumn-born children [58]. Moreover, higher schizotypal traits and a tendency of lower agreeableness were found in healthy controls born during winter months compared to those born in the remainder of the year [59], [60]. These findings suggest that the season of birth may not only affect patients with schizophrenia but as well –in a lesser degree- also healthy controls. Therefore, it would be interesting in future research to probe for an association between the season of birth, WM integrity and different psychometric measures in healthy controls and schizophrenia.

The reported WM integrity impairments in patients born in summer were in line with general findings of WM abnormalities in schizophrenia, which have predominantly been observed in left frontal and left temporal parts of the brain [61]. WM integrity alterations have also been linked to specific symptoms in schizophrenia [62]. Especially, the corpus callosum, which connects both hemispheres, has repeatedly been found to be affected in schizophrenia and was related to “hypofrontality” as well as to the dysfunctions of the heteromodal association cortex and social cognition deficits [63]–[65]. Further, decreased white matter integrity in the corpus callosum, the corona radiata and other long association fibers was found to be related to poor outcome in schizophrenia as well as to cognitive impairment [66], [67]. Another study showed that posterior regions near the right supplemental motor area were associated with motor activity in schizophrenia [8]. Moreover, white matter impairments in the uncinate fasciculus were associated with increased severity of negative symptoms and impaired memory functioning [68]. The cingulum bundles, part of the limbic system, were associated with positive symptoms in schizophrenia [69]. Additionally, the superior longitudinal fasciculus, which connects frontal and parietal areas, was reported to be related to working memory as well as to auditory hallucinations in schizophrenia [70], [71].

A number of confounding factors, including age, age of illness onset, chronicity, and medication use, may affect WM integrity [72], [73]. Apart from age correlating negatively with WM integrity in widespread regions within subjects, no other confounding variables, such as PANSS scores, CPZ, age of onset or illness duration, were found to be related to WM integrity. Moreover, a later age of schizophrenia onset was found for patients born during January and February. Nevertheless, all above mentioned variables were included as covariates in the statistical analyses in TBSS. While WM integrity alterations have consistently been reported in patients with schizophrenia, the detected differences in FA values between patients and healthy controls disappeared after correction for multiple comparisons [6]. This might be due to the inclusion of a broader schizophrenia spectrum instead of core schizophrenia or differences in the implemented methods [74]. Furthermore, urban birth which was not assessed in the current study may interact with the season of birth and thus represent a potential confounding factor [3], [75]. However, migration, minority status and drug abuse which may as well contribute to the aetiology of schizophrenia did not differ between summer and winter-born patients.

In summary, the present DTI study observed a clear and novel relationship between FA values and season of birth in patients with schizophrenia, but not in healthy controls. The alterations were located in distinct WM fibre bundles. The findings are in line with the neurodevelopmental hypothesis of schizophrenia, which suggests an early contribution to abnormal brain development.

## Supporting Information

Figure S1 **Relationship of birth date and age of schizophrenia onset.** Polar plot of birth distribution in relation to age at schizophrenia onset (circular-linear correlation, r = 0.42, *p* = 0.049). In particular, patients born in January and February showed a later age of schizophrenia onset compared to patients born in the remainder of the year.
(DOCX)

Figure S2 **Location of significant TBSS results of pair-wise differences between groups and between subgroups uncorrected for multiple comparisons.** The locations of significant FA value reductions are shown for patients relative to controls (red), for summer-born controls relative to winter-born controls (green), summer-born patients relative to summer-born controls (cyan), summer-born patients relative to winter-born controls (blue), winter-born patients relative to winter-born controls (yellow).
(DOCX)

Table S1 **Significant TBSS results of pair-wise differences between groups and between subgroups uncorrected for multiple comparisons and corrected for multiple comparisons.**
(DOCX)
