# Supplementary material for: Alterations of White Matter Integrity Related to the Season of Birth in Schizophrenia: A DTI Study
Source: PLoS One. 2013 Sep 27;8(9):e75508. doi: 10.1371/journal.pone.0075508 (PMC3785501; doi:10.1371/journal.pone.0075508)
Supplement: Figure S1 — Relationship of birth date and age of schizophrenia onset. Polar plot of birth distribution in relation to age at schizophrenia onset (circular-linear correlation, r = 0.42, p = 0.049). In particular, patients born in January and February showed a later age of schizophrenia onset compared to patients born in the remainder of the year. (DOCX) [file pone.0075508.s001.docx]

**Figure S1. Relationship of birth date and age of schizophrenia onset.** Polar plot of birth distribution in relation to age at schizophrenia onset (circular-linear correlation, r = 0.42, *p* = 0.049). In particular, patients born in January and February showed a later age of schizophrenia onset compared to patients born in the remainder of the year.
