# Supplementary material for: Alterations of White Matter Integrity Related to the Season of Birth in Schizophrenia: A DTI Study
Source: PLoS One. 2013 Sep 27;8(9):e75508. doi: 10.1371/journal.pone.0075508 (PMC3785501; doi:10.1371/journal.pone.0075508)

**Figure S2. Location of significant TBSS results of pair-wise differences between groups and between subgroups uncorrected for multiple comparisons**. The locations of significant FA value reductions are shown for patients relative to controls (red), for summer-born controls relative to winter-born controls (green), summer-born patients relative to summer-born controls (cyan), summer-born patients relative to winter-born controls (blue), winter-born patients relative to winter-born controls (yellow).


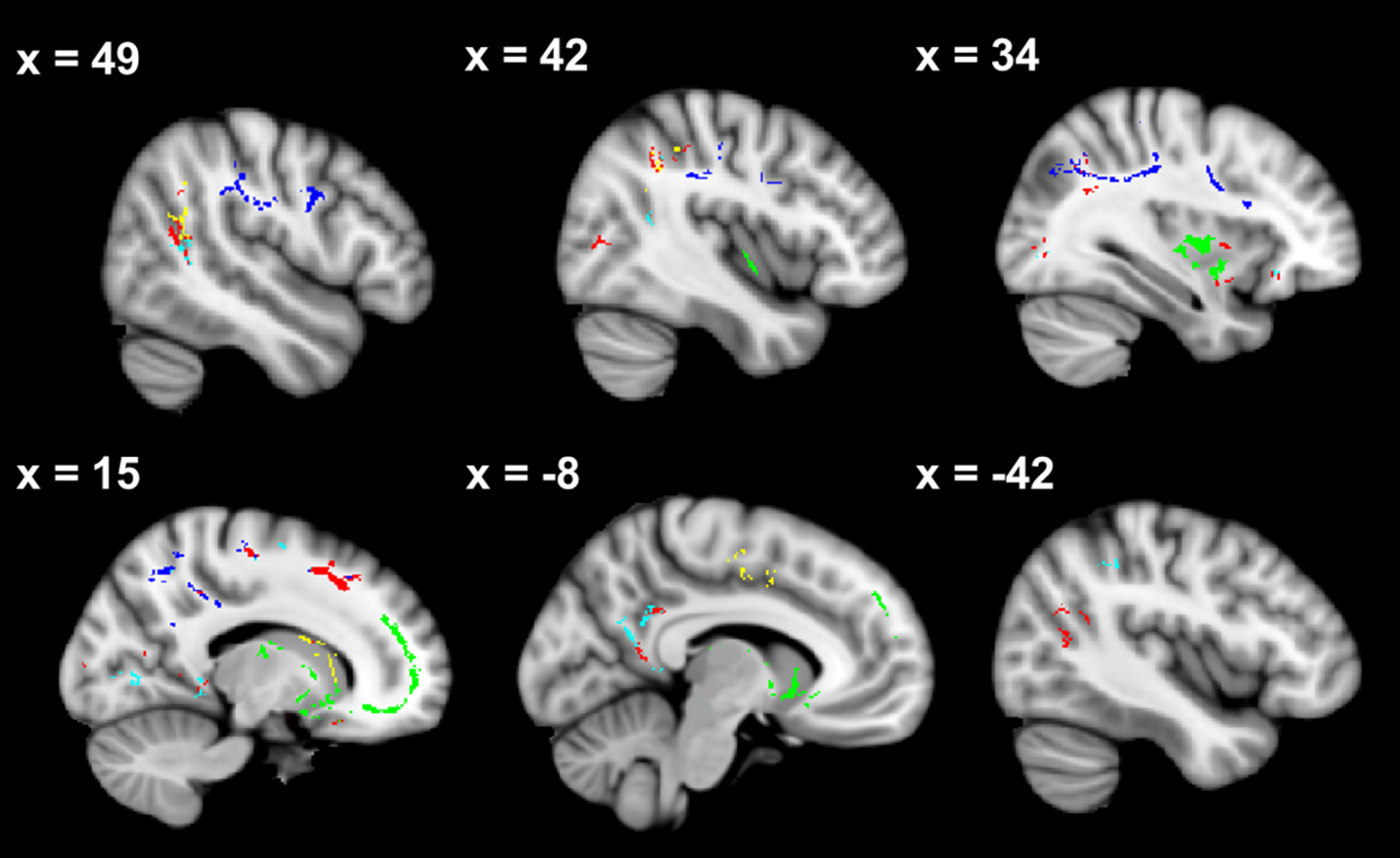

Supplement: Figure S2 — Location of significant TBSS results of pair-wise differences between groups and between subgroups uncorrected for multiple comparisons. The locations of significant FA value reductions are shown for patients relative to controls (red), for summer-born controls relative to winter-born controls (green), summer-born patients relative to summer-born controls (cyan), summer-born patients relative to winter-born controls (blue), winter-born patients relative to winter-born controls (yellow). (DOCX) [file pone.0075508.s002.docx]
