# Supplementary material for: Alterations of White Matter Integrity Related to the Season of Birth in Schizophrenia: A DTI Study
Source: PLoS One. 2013 Sep 27;8(9):e75508. doi: 10.1371/journal.pone.0075508 (PMC3785501; doi:10.1371/journal.pone.0075508)
Supplement: Table S1 — Significant TBSS results of pair-wise differences between groups and between subgroups uncorrected for multiple comparisons and corrected for multiple comparisons. (DOCX) [file pone.0075508.s003.docx]

**Table S1.** Significant TBSS results of pair-wise differences between groups and between subgroups uncorrected for multiple comparisons and corrected for multiple comparisons.

| **Contrast** | **Mean** | **SD** | **Uncorrected *p*** | **Corrected *p*** |
| --- | --- | --- | --- | --- |
| Patients <  Controls | 0.38 | 0.03 | 0.04 | 0.26 |
|  | 0.37 | 0.04 |  |  |
| Summer-born controls <  Winter-born controls | 0.42 | 0.03 | 0.02 | 0.14 |
|  | 0.46 | 0.04 |  |  |
| Summer-born patients<  Summer-born controls | 0.36 | 0.04 | 0.03 | 0.23 |
|  | 0.40 | 0.03 |  |  |
| Summer-born patients <  Winter-born controls | 0.41 | 0.04 | 0.02 | 0.12 |
|  | 0.46 | 0.04 |  |  |
| Winter-born patients <  Winter-born controls | 0.35 | 0.04 | 0.04 | 0.31 |
|  | 0.36 | 0.03 |  |  |

The means ± standard deviations (SD) of the FA values were extracted from the significant voxels (*p* uncorrected < 0.05) for each contrast separately, which may explicitly vary in the regional pattern.
